# The Working Boy
†*The Boy in Industry.* Foreword by the Right Hon. Dr. Addison, M.P. Issued by the Ministry of Munitions. 3d. H.M. Stationery Office and Fisher Unwin.


**Published:** 1917-05-19

**Authors:** 


### SOME PROBLEMS OF TO-DAY.*

### IV. The Working Boy.†

ONE of the tragedies of literature is certainly the Blue-book. Who would be seen reading one in ordinary social life? To which of us is it anything more than, as Charles Lamb neatly phrased it, "a thing in book's clothing"? Of course, if we have to "get it up" for any practical purpose we do so, just as we consult Bradshaw or a foreign dictionary. We do not "read" these useful works. Yet there are pages in Blue-books with which few novels can compare for interest, and often these are written by experts or diligent first-hand students on just those questions of the day in which we believe ourselves to be most deeply interested. There is room for a diligent writer and enterprising publisher who would place on our book-stalls a number of attractively got-up Essences of Blue-books (the title would need to be more alluring), and enable the much-needed information to reach others besides officials or secretaries of statesmen. We may welcome as perhaps a first swallow promising more such visitors the admirable little pamphlet entitled "The Boy in Industry," issued at 3d. by the Ministry of Munitions, with a foreword by Dr. Addison himself. The contents are three striking articles reprinted from the *Daily Telegraph*. Approximately 1,250,000 boys between the ages of fourteen and eighteen are, Dr. Addison tells us, employed in this country. The problem of their welfare is not a new one, but has grown much more important from causes connected with the war. There has been some crime, a good deal of unsatisfactory behaviour. The absence of fathers at the war is blamed for this, though before the war the fathers were being blamed for their lack of control. Helpful agencies, such as clubs, Boys' Brigades, Scout rallies, are too few; boys earn excessive wages, and do men's labour; hours are long in some employments, irregular in others; a spirit of unrest is in the air; the boy is suddenly left without discipline at the age when he needs it most.

Work, however, in many trades is intensely interesting in its new perfection. "Before the war we used a foot-rule," said an engineer, "now we use a micrometer." The machine is truly wonderful, but the mere human boy is himself a wonderful machine; yet we are apt to think of him only as a 'factory hand.'" He has a marvellous power of enthusiasm. Has anyone ever tried to show him the dignity of labour? He applies for work at a shop, is taken on, given a job for which he may or may not be fitted. What connection it has with the great machine which is the shop's output he may happen to learn after some years, or not at all. The practical working scheme suggested proposes a special room, a kind of schoolroom, where the younger boys could be gathered together and instructed in the various processes of the firm's undertaking. Interest and enthusiasm could so be roused and work cease to be drudgery. A significant little story is related of a boy in a munition factory, doing simple but important work, who asked for an afternoon off to "see the pictures." He had done well, and the foreman gave leave, adding, however, "But the fuses are badly needed at the Front." The boy sacrificed his coveted holiday.

The scheme suggests that entry into work should be made a matter of importance to the boy and to his parents; his enlistment in the army of industry should seem no less a thing than if he joined the army in the field. He would put more intelligence then into his choice of work, and consequently would be more likely to stick to his job and to acquire real skill, with its consequent better pay and pride of position. To that end his parents' interest needs to be enlisted also. All kinds of semi-official friends of the child do already go to visit the parents, often with a view to persuading them that it is far better for a boy to begin with small wages in a skilled trade than to take to one of the blind-alley occupations that before the war left so many young men stranded because incompetent to become unskilled and casual labourers. "'E just took to selling flowers; didn't seem to have no pertickler fancy for anything else," said a mother lately, adding, "But if he comes 'ome from France I feel 'e *will* do summat else." There is food for thought there.

Some touch between the parent and the employer of young labour would greatly help the boy. During his school years the boy's physical health was watched over, and incipient trouble could be attended to. Who will note the cause now if some ill-health is making his work indifferent or a failure? Not he himself. A quaint story is told in the pamphlet of a boy who was found to be swallowing small frogs alive. His reason was that he "had been told" the animals would eat the part of his lung which he had learnt was diseased. A graver note is sounded in the warning that the health of the race lies in the hands of the boy. Is he to be left in ignorance of the vital facts which this generation has learnt with alarm? Shall he help to propagate disease or to lift the standard of health? Will someone care enough to guide him?

His young frame needs wholesome feeding if it is to be used for labour and yet finish its development. He may have a thrifty mother who gives him a dinner to take to work. But on the whole a well-managed canteen, where food can be hot and fresh, offers the most wholesome mid-day meal. We have to remember that the lad accustomed to good regular meals has the best chance of becoming the sober man, enjoying food and not agonised by the drink appetite.

There are his earnings also, high for his age. He has not been used to have any money at all; small wonder if wild spending is a temptation! "Thrift" to the young is often an unlovely word, suggesting a joyless saving for some dull and future benefit. The joy of wise expenditure is a very real thing. Employers, we are told, sometimes look with natural vexation at empty institutes and recreation grounds. Something of the nature of a sparking-plug is needed to set the human engine to the movement of organised recreation. Leadership is the boy's loadstone. What it has meant in the field, exercised sometimes by a boy little older than himself, the world has seen of late.

The conclusion of the whole matter is convincingly set forth. The Woman Welfare Worker has already proved her value to the girl. Something of the nature of welfare work is needed for the boy, either by the appointment of a special officer possessing the qualities of a good Scoutmaster, or by the spirit informing the foremen and other authorities of the shop and carried out in some definite ways. For the subject is the *Human* Boy.

† *The Boy in Industry*. Foreword by the Right Hon. Dr. Addison, M.P. Issued by the Ministry of Munitions. 3d. H.M. Stationery Office and Fisher Unwin.

* Previous articles appeared March 31, April 7, and May 5.

